# Biophysical modulation and robustness of itinerant complexity in neuronal networks

**DOI:** 10.3389/fnetp.2024.1302499

**Published:** 2024-03-07

**Authors:** Siva Venkadesh, Asmir Shaikh, Heman Shakeri, Ernest Barreto, John Darrell Van Horn

**Affiliations:** ^1^ Department of Psychology, University of Virginia, Charlottesville, VA, United States; ^2^ Department of Computer Science, University of Virginia, Charlottesville, VA, United States; ^3^ School of Data Science, University of Virginia, Charlottesville, VA, United States; ^4^ Biomedical Engineering, University of Virginia, Charlottesville, VA, United States; ^5^ Department of Physics and Astronomy and the Interdisciplinary Program in Neuroscience, George Mason University, Fairfax, VA, United States

**Keywords:** bursting, synchronization, metastability, emergence, complexity, networks, itinerancy

## Abstract

Transient synchronization of bursting activity in neuronal networks, which occurs in patterns of metastable itinerant phase relationships between neurons, is a notable feature of network dynamics observed *in vivo*. However, the mechanisms that contribute to this dynamical complexity in neuronal circuits are not well understood. Local circuits in cortical regions consist of populations of neurons with diverse intrinsic oscillatory features. In this study, we numerically show that the phenomenon of transient synchronization, also referred to as metastability, can emerge in an inhibitory neuronal population when the neurons’ intrinsic fast-spiking dynamics are appropriately modulated by slower inputs from an excitatory neuronal population. Using a compact model of a mesoscopic-scale network consisting of excitatory pyramidal and inhibitory fast-spiking neurons, our work demonstrates a relationship between the frequency of pyramidal population oscillations and the features of emergent metastability in the inhibitory population. In addition, we introduce a method to characterize collective transitions in metastable networks. Finally, we discuss potential applications of this study in mechanistically understanding cortical network dynamics.

## 1 Introduction

Computational modeling of neuronal dynamics which incorporates mesoscopic-level connectivity features can potentially offer powerful frameworks for understanding neuronal network mechanisms (Markram et al., 2015; Kopsick et al., 2022). Despite the extensive theoretical work on the emergent dynamics in neuronal networks (Amit, 1989; van Vreeswijk and Sompolinsky, 1996; Rolls and Treves, 1997; Brunel, 2000; Gerstner and Kistler, 2002; Gerstner et al., 2014; Tsuda, 2015), a scalable network representation of key neurodynamical features at the mesoscopic level is currently lacking. A simplified model describing the complexities of between- and within-group neuronal interactions is necessary to advance our multiscale understanding of neuronal systems in typical and atypical conditions (Venkadesh and Van Horn, 2021).

An experimentally observable complexity of *in vivo* networks is the metastable attractor dynamics at the level of individual neurons. More specifically, an individual neuron can exhibit periodic bursting oscillations that transiently synchronize with the oscillations of other neurons, and these synchronizations can occur at different phases (Skaggs and McNaughton, 1996; Poe et al., 2000; Jones and Wilson, 2005; Lisman, 2005; Ego-Stengel and Wilson, 2007; Vinck et al., 2010). In other words, biological neuronal networks can realize a range of attractor states that are locally observable through different phase-locking modes between individual neurons. Such transient attractor dynamics are also referred to as metastability or itinerancy (Venkadesh et al., 2020), and this phenomenon is hypothesized to be a necessary physical property underlying the coordinated dynamics between spatially distant neuronal populations (Tognoli and Kelso, 2014).

The mathematical conditions for the emergence of transient dynamics have been previously discussed (Tsuda, 2009). More recently, conduction delays between brain areas were associated with metastable oscillations on a macroscopic spatial scale (Cabral et al., 2022). Many possible mechanisms of metastability have been proposed (Kelso, 1996; Shanahan, 2008; Brinkman et al., 2022; Hancock et al., 2023; Mackay et al., 2023). However, the biophysical features of neuronal networks underlying metastability at the level of neurons have not been fully elucidated. While a previous work reported metastable attractor dynamics among intrinsically bursting neurons (Venkadesh et al., 2020), it was unclear if metastability could also be realized in neuronal populations in which bursting dynamics are induced externally. Here, we explore the emergence and characteristics of metastability in a fast-spiking interneuron population that exhibits bursting dynamics due to driving inputs from a population of slower pyramidal neurons. We also examine the quantitative characteristics of collective transitions between attractor states realized in different metastable networks. Finally, we discuss potential applications of our work in understanding network physiology in neurodegenerative disorders.

## 2 Methods

Networks consisting of 100 pyramidal neurons and 50 fast-spiking interneurons were constructed using Izhikevich neurons (Izhikevich, 2003), where each neuron was governed by Eqs 1–3. The parameters a,b,c, and d (Eqs 2, 3) were selected to match the intrinsic dynamics of each type of neuron. Specifically, these were set to 0.02, 0.2, −65, and 8 respectively for the pyramidal neurons, and 0.1, 0.2, −65, and 2 for the fast-spiking interneurons (Izhikevich, 2003).

**FIGURE 1:**
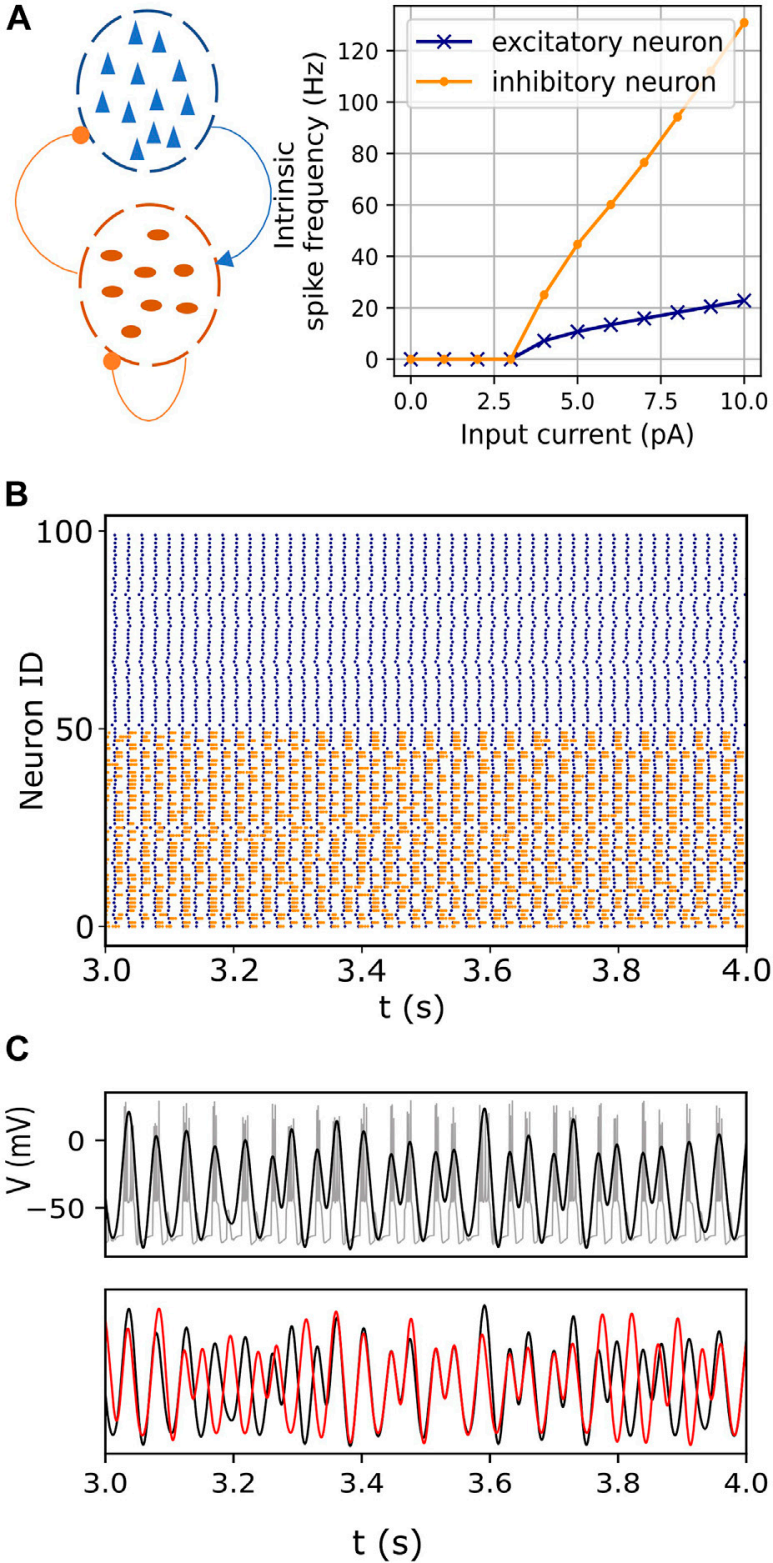
Endogenous itinerancy in fast-spiking interneurons. **(A)** *Left:* Network configuration consisting of pyramidal neurons (blue triangles) and fast-spiking interneurons (dark orange ovals). Connections indicated by the arrowhead and circles denote excitatory and inhibitory pulse-coupling, respectively. *Right:* Spiking frequencies of an isolated excitatory (pyramidal) neuron and an inhibitory (fast-spiking) neuron for various input currents. **(B)** Raster plot showing spiking activity in pyramidal (blue) and fast-spiking neurons (orange) for one second. **(C)** (top) Bursting activity (gray) in one representative fast-spiking neuron. The filtered signal, superimposed in black, captures the burst oscillations. (bottom) Burst oscillations of two representative fast-spiking neurons (black and red) showing transitions between in- and anti-phase transiently locked modes.

Network connections were configured as illustrated in Figure 1A. Inter- and intra-population connections were realized through instantiations of our networks using pairwise connection probabilities between individual neurons. We studied the network behavior for a complete range of connection probabilities. Post-synaptic effects were specified using an instantaneous pulse-coupling scheme (Eq. 4), where each presynaptic spike either increased (excitatory) or decreased (inhibitory) the postsynaptic current by the weight parameter (+0.3 for excitatory connections and −0.3 for inhibitory connections).

$$\frac{dv}{dt}=0.04v^{2}+5v+140-u+I^{ext}+I^{syn}$$ (1)

$$\frac{du}{dt}=a\left(bv-u\right)$$ (2)

$$if\;v\geq 30mV,\left\{\begin{array}{l} v=c\\ u=u+d \end{array}\right.$$ (3)

$$I_{j}^{syn}=\sum_{i=1}^{m_{j}}\delta \left(t-t_{i}\right)∙w$$ (4)

In Eq. 4, δ corresponds to the Dirac delta distribution, which equals one when a presynaptic neuron *i* spikes and is zero otherwise. $I_{j}^{syn}$ is the total synaptic current for postsynaptic neuron *j*, which receives connections from a total of $m_{j}$ presynaptic neurons. The number of neurons and the weight parameters were chosen to allow the pyramidal population to induce sustained bursting activity in the fast-spiking interneuron population for a range of external input currents in the former ( $I^{ext}$ in Eq. 1). $I^{ext}$ was always set to zero for the fast-spiking interneurons. All networks were simulated using Brian2 (Stimberg et al., 2019) for a duration of 10 s with a timestep of 0.1 m using the Euler integration method. Following the network simulation, a fifth-order low-pass Butterworth filter (Selesnick and Burrus, 1998) was applied to the voltage signal of each inhibitory neuron ( v in Eq. 1) to extract the bursting oscillations without the individual spikes (Figure 1C top). The filtered signals were standardized to have zero mean and unit standard deviation. Then, instantaneous phases of the bursting oscillations were assigned to each neuron via the Hilbert transform. Figure 1C bottom shows two representative examples.

### 2.1 Characterizing phase relationships between bursting oscillations

The basic phenomenon of interest is illustrated in Figure 1C bottom, which shows the bursting oscillations of a representative pair of neurons versus time. At first, the neurons are locked in phase, but then they transition to an out-of-phase state. After a short time, they return to the in-phase state, and then switch back to the out-of-phase state. Similar persistent behaviors were described in previous reports (Kasatkin et al., 2019; Venkadesh et al., 2020).

**FIGURE 2:**
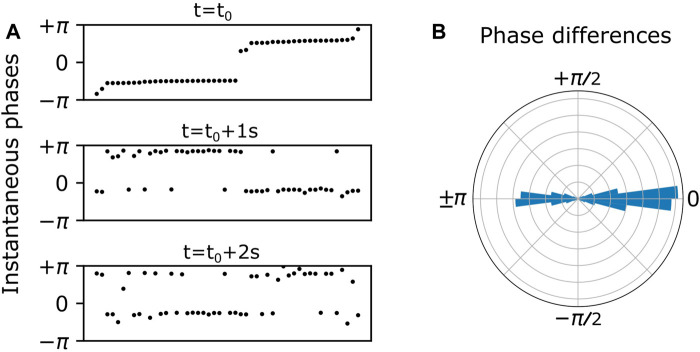
**(A)** Endogenous itinerant states characterized by phase clusters at three time points over a 2 s duration. Neurons (black dots) are ordered by their instantaneous phases in the top panel, and this ordering is preserved in the bottom two panels. Two phase clusters persist over time, and individual neurons spontaneously switch from one cluster to the other. **(B)** Persistent phase relationships between neurons are illustrated in a polar histogram by plotting the phase differences between all distinct pairs of neurons over the 2 s duration.

At the population level, the bursting phases tend to cluster and exhibit itinerant switching between clusters. This is illustrated in Figure 2. In the top panel of Figure 2A, the dots represent the instantaneous phases of all the inhibitory neurons at time $t_{0}$ , with the neurons listed horizontally according to increasing phase. We see two prominent phase clusters. In the lower panels, the neuron ordering is preserved from the top panel, and the phases are plotted for subsequent times $t_{0}+1s$ and $t_{0}+2s$ . We see that the two clusters remain. However, some neurons have hopped from one cluster to the other, exhibiting itinerant switching among cluster states.

In general, the instantaneous phases of the cluster states drift over time. In order to ignore or cancel out this drift, we calculated the phase *differences* between all possible neuron pairs over all values of discrete time. Plotting these phase differences on a polar histogram as in Figure 2B, we see phase difference clusters at zero and 180°. This is essentially a shift to a reference frame which gives a convenient and consistent way to visualize the phase clusters shown in Figure 2A.

More precisely, we assessed the nature of population-wide phase relationships among interneurons by calculating instantaneous phase differences between randomly selected neuron pairs over the entire duration (10 s). Henceforth, these phase differences will be referred to as $\phi_{j},j=1,2,\ldots ,N$ , where N denotes the number of phase difference measurements. We found that the ensemble of angles $\phi_{j}$ was organized into clusters. To characterize this, we calculated the Kuramoto-Daido order parameters $Z_{n}$ (Daido, 1996; Golomb and Hansel, 2000; Venkadesh et al., 2020) for various values of n , a positive integer:

$$Z_{n}=\frac{1}{N}\sum_{j=1}^{N}e^{in\phi_{j}}.$$ (5)

These order parameters characterize clustering patterns in the ensemble as follows. If each ensemble member is plotted as a point on the unit circle in the complex plane at its angle, $\phi_{j},$ then $Z_{1}$ is the centroid of the ensemble. Of interest is the magnitude $\left|Z_{1}\right|$ , which ranges from zero to one. If all points occur at the same angle, then $\left|Z_{1}\right|=1$ . In contrast, if the points are scattered uniformly around the unit circle, then $\left|Z_{1}\right|=0$ . Additionally, if the ensemble of points consists of uniformly spaced clusters, then $\left|Z_{1}\right|=0$ . Such uniformly spaced cluster arrangements can be identified using the order parameters $Z_{n}$ with n>1 . For example, if the phases form three ideal clusters (i.e., equally populated clusters 120° apart), then $\left|Z_{1}\right|=\left|Z_{2}\right|=0$ , but $\left|Z_{3}\right|=1$ . In fact, for ideal states consisting of c uniformly spaced and equally populated clusters, $\left|Z_{jc}\right|=1$ for any integer j≥1 and is zero otherwise. To correct for this redundancy, we defined a real-valued quantity $G_{n}$ as in Eq. 6, such that $G_{n}$ is close to 1 only when the ensemble approximates an ideal n -cluster state.

$$G_{n}\left.=\right|Z_{n}\left|\times \right.\prod_{k=1}^{n-1}\left(\left.\left.1-\right|Z_{k}\right|\right)$$ (6)

### 2.2 Characterizing exogenously induced transitions

To assess the nature of transitions between clustered states in different metastable networks, the following steps were carried out: A given network was simulated twice with identical initializations. The first simulation was used to establish the baseline instantaneous clustering of neurons, based on their instantaneous phases, at any given time. In the second simulation, a small group of randomly selected interneurons were simultaneously perturbed by single spikes at a certain time. Perturbations were applied via a single spike from an external pyramidal neuron. More specifically, each perturbed interneuron received a single spike that increased its voltage level by a value specified by the perturbation weight. We studied the effect of such perturbations for various numbers of perturbed neurons and for a range of perturbation weights. The perturbations caused some of the neurons to subsequently change their cluster assignments relative to the baseline.

Phase cluster assignments of neurons were obtained using Gaussian mixture models (GMM) (Pedregosa et al., 2011). The GMM group the instantaneous phases of neurons into n clusters, where n is identified by Eq. 6. This method assumes that the clusters of instantaneous phases of neurons are normally distributed. The GMM models are initialized with randomly selected phases. A total of 100 iterations were performed for GMM fitting.

To quantify the effect of the perturbation, we calculated the cluster assignment of neurons at a given time in the baseline simulation, and compared this arrangement with how the neurons were distributed among the clusters at the corresponding time in the second simulation (with the perturbation). We measured the similarity of these two clustering arrangements of the neurons using Eqs 7–9.

First, we calculated the well-known “Rand Index” in Eq. 7 (Rand, 1971), where g and ℎ are the numbers of pairs of neurons with the same and different cluster assignments across the two groupings that are being compared, and $nC_{2}$ denotes the number of possible combinations of neuron pairs. The Rand Index (RI) is defined such that it equals one for two identical arrangements of neurons among the clusters. We then calculated the Adjusted Rand Index (ARI), defined in Eq. 8 (Rand, 1971; Hubert and Arabie, 1985; Steinley, 2004). This is a variation of RI that accounts for chance, ensuring that the ARI for two random groupings of a set of neurons is zero while remaining equal to one for identical arrangements. This is achieved by estimating an expected RI ( $E\left(RI\right)$ in Eq. 8) using random permutations of cluster assignments. The result was the ARI as a function of time, measuring the ongoing effects of the perturbation on the clustering arrangements.

$$RI=\frac{g+h}{nC_{2}}$$ (7)

$$ARI=\frac{RI-E\left(RI\right)}{\max\left(RI\right)-E\left(RI\right)}$$ (8)

$$CR=1-\frac{1}{M}\sum_{i=1}^{M}{ARI}_{i}$$ (9)

We then defined the population’s collective response CR as in Eq. 9 to characterize the extent to which the network (as a whole) generically responds to exogeneous perturbations of a fraction of its elements. The ARIs were measured in 50 simulation trials, each with different realizations of the random connections ( M=50 in Eq. 9), and each with a random subset of perturbed interneurons and a random time (>0.5 s) at which the perturbation was delivered. The observed average change in the ARI following the perturbation denoted the effect of the perturbation. For instance, if there is no change to the network state following the perturbation, then all the ARIs are one and the collective response is zero. On the other hand, if a perturbation results in rearrangements of the neurons among the clusters that are equivalent to completely random cluster assignments compared to the baseline condition, then all the ARIs are zero and the collective response is one. Finally, the magnitude of the collective response was examined in metastable networks for various numbers of perturbed neurons in the range [1, 20] and for various perturbation weights in the range [0.1, 1]. It is worth mentioning here that while each presynaptic spike influences the activity of a postsynaptic neuron in a discrete manner [see Eq. 4], the collective response aims to characterize activity that is spatiotemporally coarse-grained (i.e., transitions that occur among a group of neurons on a relatively larger timescale than that of individual spikes).

## 3 Results

The network model incorporates a significant difference in the spiking time scales of the pyramidal and fast-spiking interneurons. The slower excitatory drive from the pyramidal neurons modulates the fast-spiking dynamics of the interneurons, resulting in bursting oscillations in the latter (Figures 1B, C). The inhibitory interactions between these bursting neurons then lead to itinerant metastable behavior. That is, ongoing endogenous transitions occur between different phase-locked (attractor) states. (Figure 1C, also see Section 2.1).

### 3.1 Interneuron population phase clusters are modulated by the excitatory frequency

**FIGURE 3:**
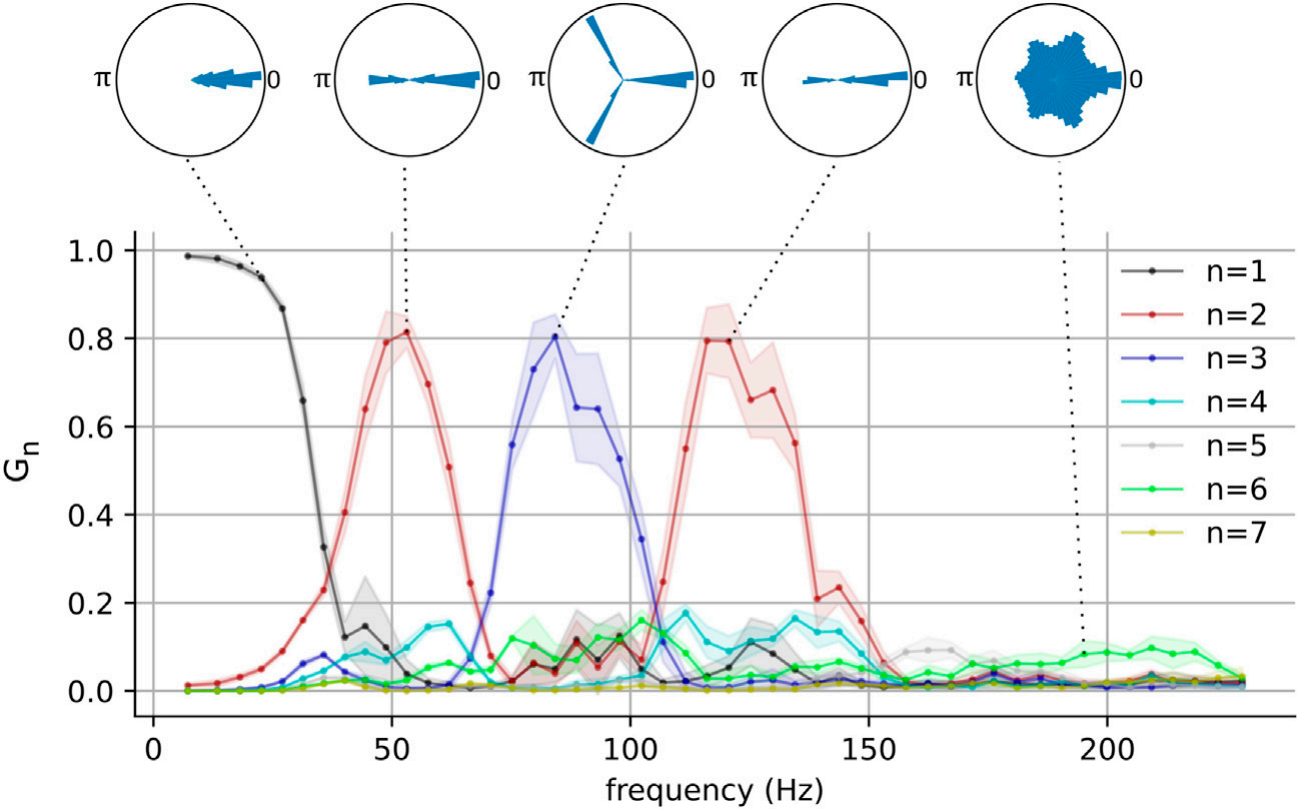
The network-wide stability and the number of phase clusters in the fast-spiking interneurons as functions of pyramidal neuron frequency for one representative network. Polar histograms show phase differences $\phi_{j}$ for all pairs of neurons in representative networks. Pyramidal neuron frequency was computed by taking the inverse of the average population interspike intervals. For each frequency, $G_{n}$ values were computed by randomly sampling 10 sets of 100 pairs of interneurons. Solid lines and shaded areas represent means and standard deviations respectively.

The frequency of pyramidal neuron spiking is determined by the value of $I^{ext}$ . To examine the effect of this driving frequency on the characteristics of metastability in the fast-spiking interneuron network, we systematically varied $I^{ext}$ and measured the distribution of phase differences ( $\phi_{j}$ ) between pairs of interneurons as described in Methods Section 2.1. Figure 3 shows a plot of $G_{n}$ (*n* = 1, 2, … , 7), which quantifies the degree to which the interneuron phase differences group into *n* clusters, versus the pyramidal neuron spiking frequency (*f*) for one representative network with E-I and I-I connection probabilities of 0.7 and 0.4 respectively. Above the plot are polar histograms of the phase differences ( $\phi_{j}$ ) for selected *f*’s. At low driving frequency, all the inhibitory neurons lock in-phase, and thus their phase differences are close to zero. Correspondingly, $G_{1}$ is high and $G_{2}$ through $G_{7}$ are low. Interestingly, as the driving frequency increases beyond ∼25*Hz*, clusters appear in the phase difference distribution. We find that a well-defined state of two clusters emerges near *f* = 48 Hz, and a three-cluster state emerges near *f* = 77 Hz. As the driving frequency is further increased, the pattern reverses and another well-defined state of two clusters appears near *f* = 117 Hz. Beyond *f* = 140 Hz, all $G_{n}$ are small and the phase difference distributions become more uniform. (see Supplementary Figure S3 for a visualization of cluster-switching in three-cluster states).

These results show that metastable attractors emerge in the interneuron population when it is appropriately driven by the pyramidal population, and that the nature of these metastable attractors is modulated by the driving frequency. It is interesting to note that the emergence of these clustered states occurs for driving frequencies within the gamma and the high-gamma ranges. This behavior was also present in a larger population of interneurons (see Supplementary Figure S1). Pyramidal neurons have indeed been observed to fire at these frequencies (O’Keefe and Recce, 1993; Dragoi and Buzsáki, 2006; Ray et al., 2008), although such gamma cycles have been observed to be modulated by slower theta ones. Additionally, although it is typical for neurons to generally exhibit aperiodic firing *in vivo*, selectively recruited pyramidal neurons are known to fire bursts of action potentials in a periodic manner and the phases of these bursts are strongly correlated with an organism’s behavior (O’Keefe and Recce, 1993; Dragoi and Buzsáki, 2006).

**FIGURE 4:**
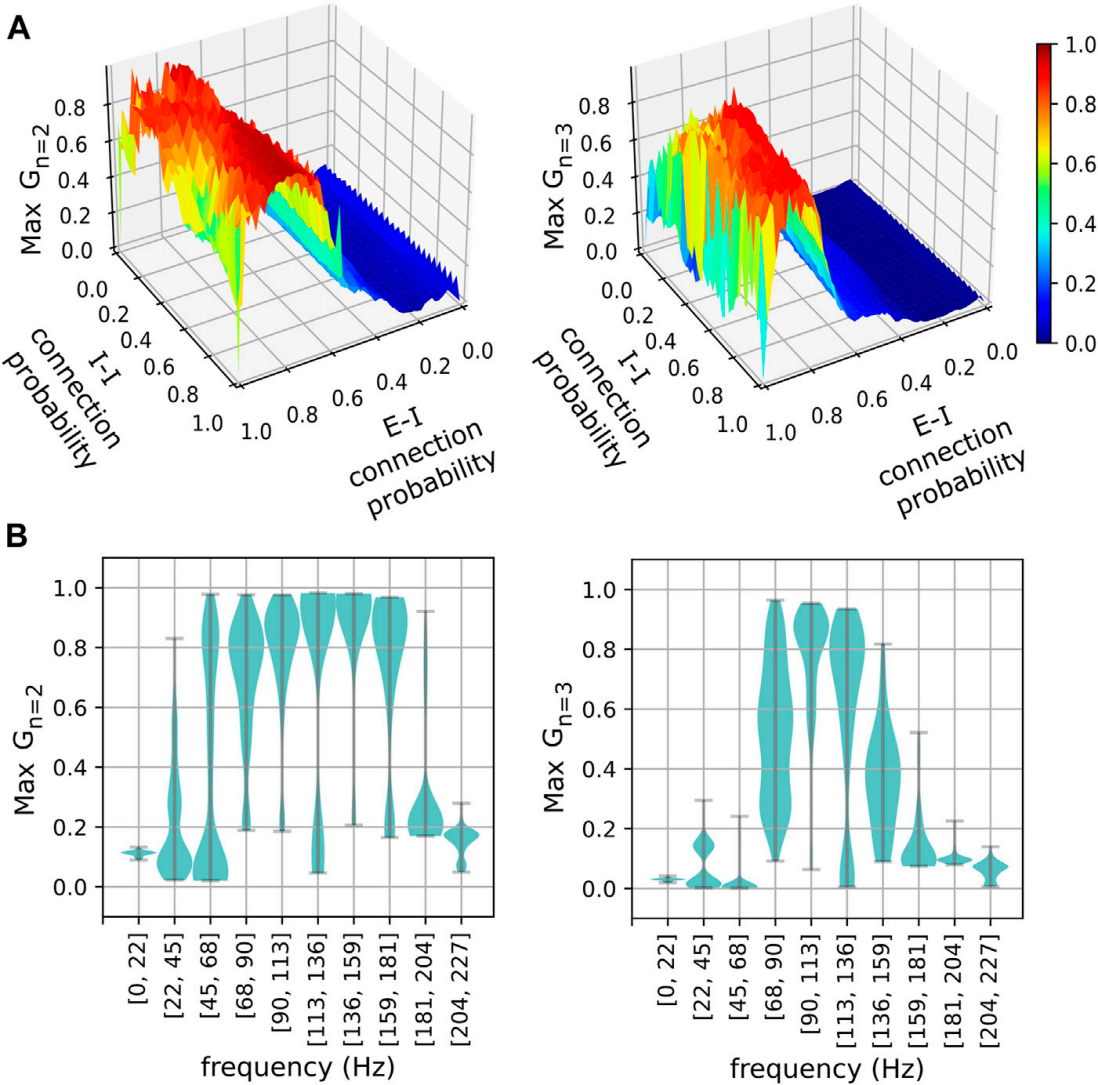
The stability of phase clusters versus network connectivity and pyramidal neuron frequency. **(A)** Stabilities of the maximally stable two-cluster (left) and three-cluster (right) states realized in networks for various excitatory-inhibitory (E-I) and inhibitory-inhibitory (I-I) connection probabilities. **(B)** Distributions of maximum phase cluster stability are plotted against their respective frequency bins of pyramidal neurons for two-cluster (left) and three-cluster (right) states. Width indicates the density of points for a given maximum stability.

The phase-clustering phenomena described above were consistently observed in networks with different connectivity profiles realized using a range of connection probabilities (Figure 4). We analyzed networks instantiated using 50 × 50 values of equally spaced inter- and intra-group connection probabilities in the range [0, 1]. The $G_{2}$ and $G_{3}$ versus frequency curves were computed as described before for each of the 2,500 networks. Figure 4A shows the maximum of $G_{2}$ (left) and $G_{3}$ (right) across frequencies for each network. These measures were sensitive to the E-I connection probabilities and relatively less sensitive to the I-I connection probabilities. Plotting these measures against the frequency of the excitatory neurons (Figure 4B) revealed that the stable phase clusters emerged approximately within the range [45 *Hz*, 180 *Hz*]. Our results suggest that the interneuron population’s emergent attractors depend primarily on the E-I connectivity and the frequency of the driving excitatory oscillations. There are robust gradients (Daoud et al., 2023) towards maximally stable interneuron phase cluster arrangements along the dimensions of pyramidal frequency and E-I connection probability (Figures 3, 4).

Although the $G_{n}$ values were relatively less sensitive to the inhibitory I-I connectivity, the I-I connections are necessary for the itinerant behavior to emerge. It is possible for multiple non-itinerant phase clusters to appear when both the I-I connection probability is zero *and* the E-I connection probability is less than 1 (Figure 4A). Pairwise connection probabilities less than 1 introduce small differences in the number of connections among neurons. This may result in different excitation levels in some inhibitory neurons causing a phase shift. However, it is important to note that such cases do not exhibit switching between different clusters as there are no inhibitory interactions (See Supplementary Figure S2). Additionally, when the I-I connection probability is zero and E-I connection probability is *equal* to 1.0, all inhibitory neurons achieve identical excitation levels. In this scenario, the non-itinerant 2-cluster state disappears, which can also be observed in Figure 4A.

### 3.2 Collective response characteristics of metastable networks

**FIGURE 5:**
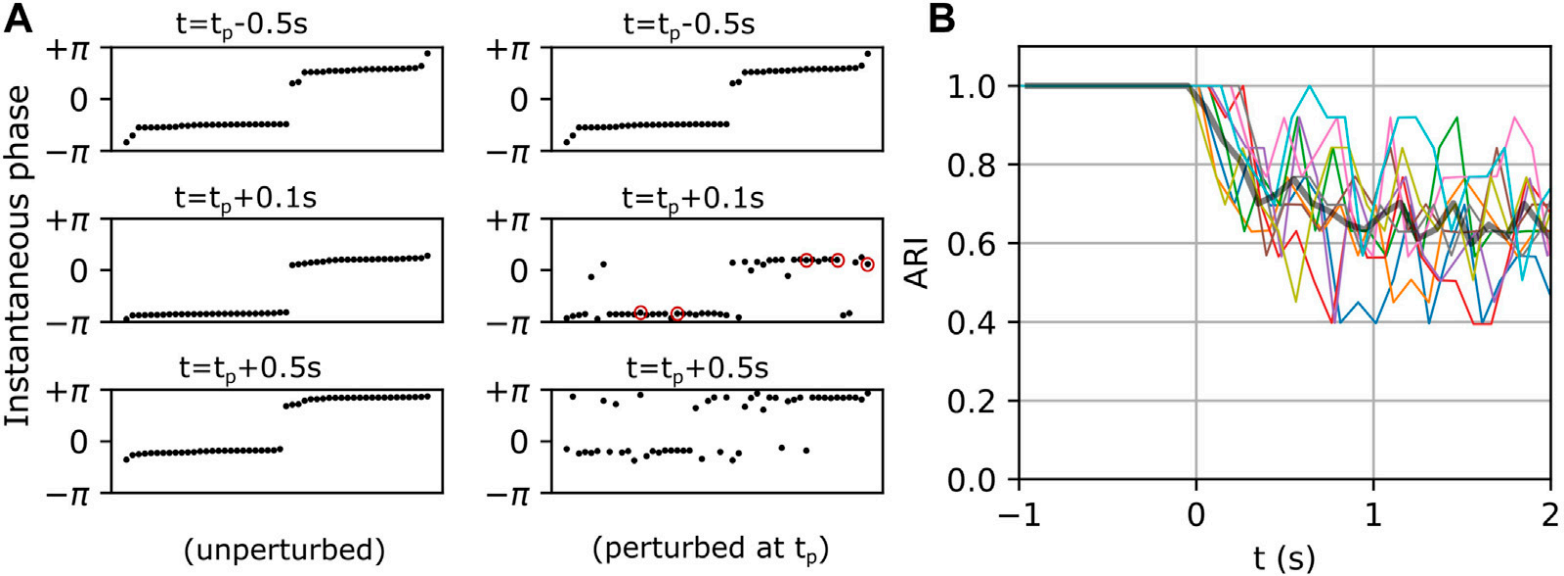
Exogenous transitions in the fast-spiking interneuron population. **(A)** Left and right panels show the temporal evolution of a network in unperturbed and perturbed conditions respectively. Neurons (black dots) in the left panels are sorted by their instantaneous phases at each t , and their order is preserved in the right panels to show the divergence of states caused by perturbation. $t_{p}$ denotes the time of perturbation, and red circles in the right middle panel denote perturbed neurons. **(B)** Ten representative ARI curves (color) computed by comparing cluster assignments of neurons between unperturbed and perturbed conditions. The black curve represents the average. Here, t = 0 corresponds to the perturbation times $t_{p}$ .

The multi-cluster states described above exhibit spontaneous and endogenous itinerant dynamics (see Figures 1C, 2). Motivated by the fact that an organism adapts its internal state to the environment via external stimuli, we sought to measure how a metastable state would be affected by an external perturbation. As described in Methods Section 2.2, the networks were simulated twice with identical initializations. The first simulation established the baseline dynamics as shown in Figure 5A (left column). The panels on the left show the instantaneous phases of individual neurons in the interneuron population at three instants of time, with the neurons ordered along the horizontal axis by increasing phase. Note that the neurons were reordered in each left panel in Figure 5A in order to mask the endogenous itinerant behavior (see Figure 2 for endogenous itinerancy). In the second simulation, an external perturbation was applied. Five randomly selected interneurons each received a single spike at time $t_{p}$ . The results are shown in the right column of Figure 5A. The time of perturbation occurred between the top and middle panels. Note that the neuron orderings in each panel of the left column are preserved in the corresponding panels of the right column. Thus, any differences in the illustrated cluster assignment of neurons are due exclusively to perturbation. Additionally, the transitions are collective in the sense that they occur in many non-perturbed neurons immediately following the perturbation (Figure 5A—right). Note that the perturbed neurons are denoted by red circles in Figure 5A (right middle panel), and the cluster-switching can be observed in a few non-perturbed neurons at $t_{p}+0.1s$ . This highlights the synergy of interacting elements in metastable networks. Collective transitions may be characterized by treating network states as multiple instantaneous phase clusters of neurons at any given time and subsequently comparing the cluster assignments of neurons between unperturbed and perturbed conditions using the ARI (Figure 5B). For example, when $t<t_{p}$ , the two simulation conditions (left and right panels of Figure 5A) show identical cluster arrangements of neurons resulting in ARI values of 1. However, immediately following perturbation ( $t>t_{p}$ ), the network states begin to diverge between the two simulation conditions resulting in ARI values less than 1 (Figure 5B).

We examined the magnitude of collective responses (CR, Eq. 9) in three representative metastable networks that showed high magnitudes of stability in Figure 3: the networks of the two-cluster state that emerged near *f* = 48 Hz, the three-cluster state that emerged near *f* = 77Hz, and the two-cluster state that emerged near *f* = 117 Hz. For each of these three networks, we varied the number of perturbed neurons from 1 to 20 to characterize their collective responses. Additionally, we examined the effect of the perturbation weight (see Section 2.2) by varying it from 0.1 to 1.0, while the number of perturbed neurons was set to a constant value of 5.

**FIGURE 6:**
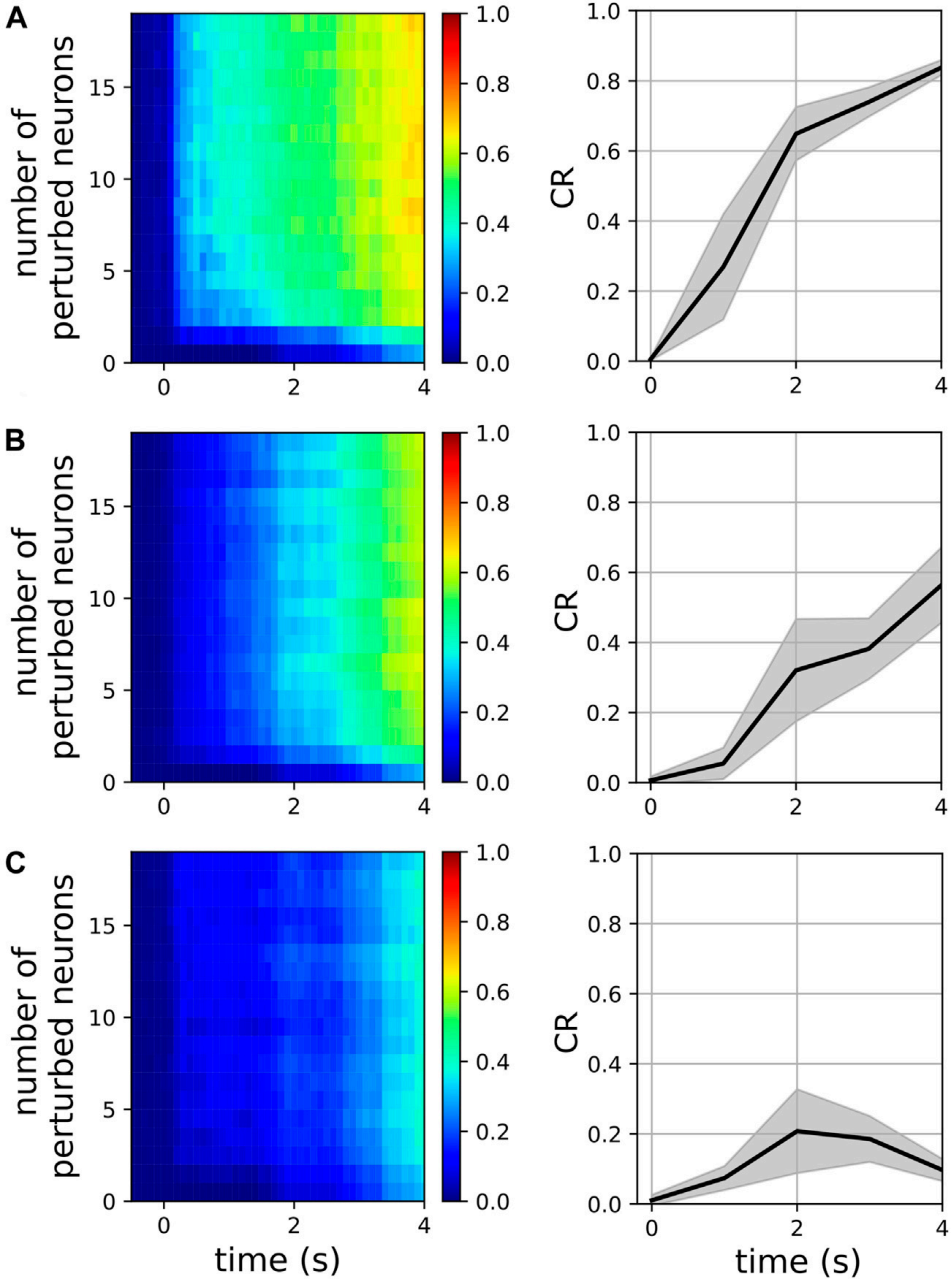
Magnitude of collective response (Eq. 9) characterized by the exogenous transitions in three representative networks for various numbers of perturbed neurons when the perturbation weight is 0.3 (left panels) and for various perturbation weights in the range [0.1, 1.0], when the number of perturbed neurons is 5 (right panels). In the right panels, solid lines and shaded regions represent means and standard deviations, respectively, across perturbation weights. The pyramidal driving frequencies used are 48 Hz **(A)**, 77 Hz **(B)**, and 117 Hz **(C)**; these correspond to two, three, and two cluster states in the interneuron population, respectively (see Figure 3).

Figure 6 shows the CR magnitudes in the three representative networks mentioned above. When only a single neuron was perturbed, the CR remained close to zero, although the perturbed networks began to show small deviations from their respective unperturbed networks at $t>t_{p}+2s$ . This was observed in all three cases (Figure 6—left panels). As the number of perturbed neurons was further increased, the networks that were sampled near *f* = 48 Hz (two-cluster state) and *f* = 77 Hz (three-cluster state) began to elicit stronger and faster responses. However, their responses reached a plateau when the number of perturbed neurons increased beyond 5 (Figures 6A, B). In general, the network that was sampled near *f* = 48 Hz was the most responsive to perturbations (Figure 6A). On the other hand, the network that was sampled near *f* = 117 Hz (two-cluster state) continued to elicit only weak responses as the number of perturbed neurons increased (Figure 6C). These observations were consistent when the perturbation weights were increased from 0.1 to 1.0 (Figure 6—right panels). Thus, the CR provides a useful metric to characterize the responsiveness of metastable networks by inducing phase cluster-switching via small perturbations. It is worth noting that *f* directly affects the inter-burst intervals in interneurons and consequently shapes their multi-periodic trajectories in the phase space. Taken together, these results suggest that the metastability realized in interneuron populations may arise from mechanisms (Ott et al., 1993; Sommerer and Ott, 1993; Tsuda, 2009) that evolve depending on (a) their connectivity with excitatory neurons and (b) the frequencies of excitatory oscillations within a cortical region.

## 4 Discussion

Understanding the emergent dynamical complexities in a network in terms of its constituent mechanisms remains an important goal. Here, we studied the emergent phenomenon of itinerancy, an instance of metastability, in spiking neuronal network models that represent cortical local circuits. Metastability is characterized by the coexistence of integrated (synchronized states) and segregated (transitions between synchronized states) behaviors in a system of interacting elements (Tognoli and Kelso, 2014). In this study, we characterized the extent to which neurons are synchronized using the Kuramoto-Daido order parameters for various numbers of phase clusters (Eqs 5, 6). We examined the extent to which neurons collectively transition between different synchronized states using a novel metric (Eq. 9). We wish to note that many metrics of metastability in the literature aim to quantify the extent to which both synchronized and desynchronized states coexist in a system of interacting elements. While these metrics provide useful signatures of metastability, they do not capture the synergistic nature of transitions in metastable systems. In this study, once the number of phase clusters in a network was identified using $G_{n}$ (Eq. 6), the metric CR (Eq. 9) was used to characterize the synergy of transitions between the phase clusters by applying small perturbations.

In a previous study (Venkadesh et al., 2020), we showed that networks of complex periodic oscillators such as intrinsically bursting neurons can realize chaotic itinerancy (Tsuda, 2015). Chaotic itinerancy is an instance of metastability that is realized by the transitions between different basins of attraction through heteroclinic orbits (Hancock et al., 2023). In our current work, we studied how the intrinsic biophysical features of a spiking neuronal network are quantitatively related to metastability using a generalized model of cortical intraregional interactions. We specifically studied how the neuron-level spiking frequencies are associated with the emergent metastability, and how these relationships are governed by the underlying excitatory and inhibitory neuronal connectivity.

We showed that (1) driving input from the pyramidal population to the fast-spiking neurons caused bursting in the latter (see (Pinsky and Rinzel, 1994) for an investigation of the mechanisms of bursting), and that this led (due to inhibitory coupling) to itinerancy in the bursting phase relationships in the fast-spiking population; (2) the complexity of the itinerancy (in the form of the number of clusters) was modulated by the pyramidal driving frequency; (3) this behavior was robust with respect to the network connection probabilities as long as the E-I probability was large enough; (4) the different itinerant states had different responsiveness to perturbations.

It is worth comparing our current model and observations with those of our previous study (Venkadesh et al., 2020). Our previous model of itinerant complexity, which was constructed using only an inhibitory population of intrinsically bursting neurons is simpler than our current model, which includes both an excitatory and an inhibitory population. The construction of our current model was motivated by several considerations: First, intrinsically bursting neurons are biologically rare. For instance, less than 15% of inhibitory neurons are intrinsically “bursting” (multi-periodic) in the rodent hippocampus, and the remaining neurons are intrinsically “spiking” (single-periodic) (Komendantov et al., 2019). Our current model approximation includes slower excitatory and faster inhibitory neurons, both of which are intrinsically single-periodic, and this circuit configuration is ubiquitous in the cortex. Furthermore, as we showed in our previous study, the intrinsically single-periodic neural populations, by themselves, are unable to realize itinerant complexity. On the other hand, the current model describes mechanisms by which itinerant complexity could be realized in such simpler inhibitory neurons that vastly outnumber their intrinsically bursting counterparts in the cortex. Taken together, our studies support the idea that the same dynamics could emerge from multiple network configurations in the cortex (Friston and Price, 2003). Secondly, our previous model did not show that the number of phase clusters within an inhibitory population could change depending on the frequency. In fact, our previous study reported emergent itinerancy at different bursting regimes of the model, where 3-cluster states that only differed in their magnitudes of stability were observed. On the other hand, our current model shows, in addition to the magnitude of stability, the number of phase clusters can also change within an inhibitory population depending on the frequency of excitatory drive. It is worth noting that many intrinsically bursting neurons have a narrow regime for bursting (Komendantov et al., 2019). In other words, the range of excitatory inputs for which an intrinsically bursting neuron actually bursts (as opposed to spike) is narrow. On the other hand, the externally induced bursting is observable for a wide range of excitatory inputs as we have shown in our current study. Finally, our current model suggests that the inhibitory population’s itinerancy could be modulated by electrically stimulating excitatory neurons such as the pyramidal neurons. This is especially important in the context of neuromodulation (Luan et al., 2014; Krishna et al., 2018; Cole et al., 2022). Targeting pyramidal neurons for neuromodulation is currently more feasible than targeting regionally local inhibitory neurons. Because pyramidal neurons project their axons over long distances and their connectivity can be noninvasively mapped using neuroimaging modalities, they are highly feasible targets for neuromodulation. On the other hand, an inhibitory neuronal population’s connectivity is generally restricted to a cortical region, and current neuromodulation methods lack spatial specificity to selectively target such regionally local neuronal populations. Overall, while our previous model was computationally more compact than our current model, this compactness traded off its practical applicability. On the other hand, our current model, while still scalable, has broader applicability and provides a framework for future studies to make testable predictions as discussed in the final paragraph of this section.

The number of connections an inhibitory neuron receives is a crucial parameter that affects the emergence of metastable clusters. Our current model suggests that excitatory connections alone are insufficient to allow the emergence of metastable clusters in an inhibitory population (Supplementary Figure S2). Similarly, inhibitory connections alone are insufficient to allow the emergence of metastable clusters in “non-intrinsically bursting” neurons as we have shown in our previous study. Therefore, it is an appropriate balance between the excitatory and inhibitory connections that allows the emergence of metastable behaviors in our current model. We have shown that such optimal balance could be achieved for various connection probabilities (Figure 4A) and for a larger neuronal population (Supplementary Figure S1). Importantly, the frequency bins at which the 2-cluster and the 3-cluster states achieve maximal stability are consistent across various connection probabilities (Figure 4B) and for a larger neuronal population (Supplementary Figure S1). It is worth mentioning that these frequency bins are characteristic of networks with the specific neuron types considered in our model. These neuron types differ in their intrinsic spiking frequencies (Figure 1). Additionally, the diversity of cortical neuronal populations is not only revealed in their intrinsic spiking frequencies, but also in other temporal features such as delayed spiking, where a neuron elicits spikes following a brief period of quiescence for excitatory inputs. There are many neuronal populations that exhibit such delayed spiking behaviors (Komendantov et al., 2019). This is an important consideration in light of the fact that many studies associated connection delays with emergent metastability (Cabral et al., 2022; Hancock et al., 2023). Future work will investigate if and how the characteristic frequency bins reported in this study are affected by including additional inhibitory neuronal populations with distinct intrinsic spiking profiles and by incorporating a range of connection delays.

The spiking neuronal network models examined in this paper provide compact mesoscopic scale approximations of regional collective dynamics that capture a crucial biological complexity at the level of individual neurons. A limitation of the current work is that it only considered two types of neuronal populations, whereas intraregional neuronal circuits consist of populations with diverse frequency profiles (Komendantov et al., 2019). Nevertheless, the current work demonstrates that the emergent metastability in an interneuron population depends on their connectivity with excitatory neurons and the frequency of the excitatory drive. Their relationships are revealed in the $G_{n}$ curves that show robust gradients (Daoud et al., 2023) along the dimensions excitatory-inhibitory connection probability (Figure 4A) and the frequency of the excitatory drive (Figures 3, 4B). Thus, a numerical optimization using $G_{n}$ as an objective function can, in principle, estimate optimal intraregional connectivity configurations among groups of neurons with heterogenous frequency profiles.

A previous study reported clustering behaviors in a network of excitatory and inhibitory neurons (Stefanescu and Jirsa, 2008). The authors investigated cluster transitions of neurons under various conditions. It is worth noting that they reported several instances of static clusters in parameter space, where cluster transitions occurred when changing the network parameters such as the connection weights. However, endogenous transitory phenomena have been previously described by Ichiro Tsuda and collaborators using the framework of chaotic itinerancy (Tsuda, 2001; Tsuda et al., 2004). We wish to note that the novelty of our work is in the (a) description of itinerancy that occurs at the level of bursting oscillations, which was motivated by the experimental observations of this phenomenon (see Section 1), (b) association of the emergence of such itinerancy with the underlying biophysical mechanisms, most notably, the intrinsic differences in the membrane excitability levels between the excitatory and inhibitory neural populations, (c) reliable modulation of the itinerant behavior in inhibitory neurons via excitatory neurons, and (d) formulation of a method to characterize the synergy of neural transitions between multiple synchronized states. Furthermore, the Hindmarsh-Rose bursting neurons employed in (Stefanescu and Jirsa, 2008) did not show multiple phase clusters for a single network configuration. The phases of individual bursting neurons were uniformly scattered around a mean phase. The absence of multi-clustered states in their bursting neurons may be because they neglected the connections within the inhibitory subpopulation. In the present model, the bursting neurons not only show multiple distinct phase clusters for a single network configuration, but they also exhibit endogenous cluster transitions without changing the network parameters. It may also be worth pointing out the differences between their network model and the one presented in this study: (1) In (Stefanescu and Jirsa, 2008), the excitatory and the inhibitory neural subpopulations did not differ in their intrinsic dynamics. Their model assumed that in a small cortical volume, the factors affecting the membrane excitability will be similar for all neurons. However, recent efforts to comprehensively characterize the intrinsic physiology of neurons revealed enormous diversity in neural subpopulations even within a small cortical volume (Markram et al., 2015; Komendantov et al., 2019). Motivated by this fact, our model included excitatory and inhibitory subpopulations with sharply different intrinsic excitability levels, which are revealed in their current-frequency curves (Figure 1A). (2) In (Stefanescu and Jirsa, 2008), the connections within the inhibitory subpopulation were neglected. However, connectivity within the inhibitory subpopulations exist in the cortex (Wheeler et al., 2024), and it is an important characterizing feature in data-driven simulations of cortical circuits (Kopsick et al., 2022). As we have shown in this study, connections within the inhibitory subpopulation are crucial for the emergence of itinerant dynamics. (3) Finally, their model utilized a global coupling scheme for the network architecture, where every neuron is connected to every other neuron. In our model, we probabilistically realized connections between neurons, and we explored the network dynamics for the full range of connection probabilities. It is worth noting that cortical neurons are not globally connected, and connection probabilities are important parameters in data-driven simulations of cortical circuits (Markram et al., 2015; Kopsick et al., 2022).

Additionally, the models and the methods of analysis presented in this paper are easily scalable to study interregional interactions in the brain. Empirically estimated anatomical connectivity between brain regions provides useful information for modeling interregional metastability. Note that while the current work suggests a link between two temporal scales (i.e., timescales of neuronal spiking and attractor transitions) of network dynamics, modeling interregional interactions using their anatomical connectivity will further provide a venue to study metastable brain dynamics at a macroscopic spatial scale. Such models are useful mathematical tools that will enable a multiscale understanding of cortical dynamics and illuminate neuronal network mechanisms of pathophysiology in neurological disorders. As an example, we briefly discuss how a brain-wide analysis of metastable dynamics could be useful in elucidating the pathophysiology of Parkinson’s disease (PD). The structural degeneration of cortical and subcortical connectivity is a key factor underlying the motor and cognitive deficits in PD (Cochrane and Ebmeier, 2013; Atkinson-Clement et al., 2017; McGregor and Nelson, 2019). However, the mechanisms by which the structural degeneration alters the brain-wide dynamics in PD are not well understood. A whole-brain model of metastable dynamics constrained by the PD-affected anatomical connectivity can potentially delineate such mechanisms. For instance, amplified synchronization in beta frequency (8 Hz–30 Hz), which has been reported in the cortical and basal ganglia circuits of PD subjects, is hypothesized to contribute to PD symptoms (Hammond et al., 2007; Brittain et al., 2014; McGregor and Nelson, 2019). In particular, longer episodes of beta oscillations in the subthalamic nucleus (STN) (Deffains et al., 2018) and its higher synchronization with the cortical supplementary motor area (SMA) were associated with the freezing of gait in PD (Toledo et al., 2014). It may be hypothesized that the freezing of gait in PD is contributed by a reduction in synergistic transitions between different attractor states realized in these areas. The increased beta synchronization between STN and SMA observed in PD, and the hypothesized reduction in attractor transitions can be validated in whole-brain models by examining their stability (Eq. 6) and collective response characteristics (Eq. 9) respectively. Furthermore, by selectively targeting frequency bands for manipulation, one can investigate causal relationships between specific frequencies of oscillations and the dynamics of transitions between attractor states realized across multiple timescales. Such models could also enable the identification of network mechanisms for restoring optimal brain dynamics in PD and other neurological disorders via neuromodulation.

## Data Availability

The datasets presented in this study can be found in online repositories. The names of the repository/repositories and accession number(s) can be found below: https://github.com/sivaven/Transient-Synchronization.
