# Supplementary material for: Biophysical modulation and robustness of itinerant complexity in neuronal networks
Source: Front Netw Physiol. 2024 Mar 7;4:1302499. doi: 10.3389/fnetp.2024.1302499 (PMC10954887; doi:10.3389/fnetp.2024.1302499)
Supplement: Supplementary file 1 [file DataSheet1.DOCX]

Supplementary Material

**Biophysical modulation and robustness of itinerant complexity in neuronal networks**

Siva Venkadesh, ^*^ Asmir Shaikh, Heman Shakeri, Ernest Barreto, John Darrell Van Horn

*** Correspondence:** Corresponding Author: siv30@pitt.edu

## Supplementary Figures


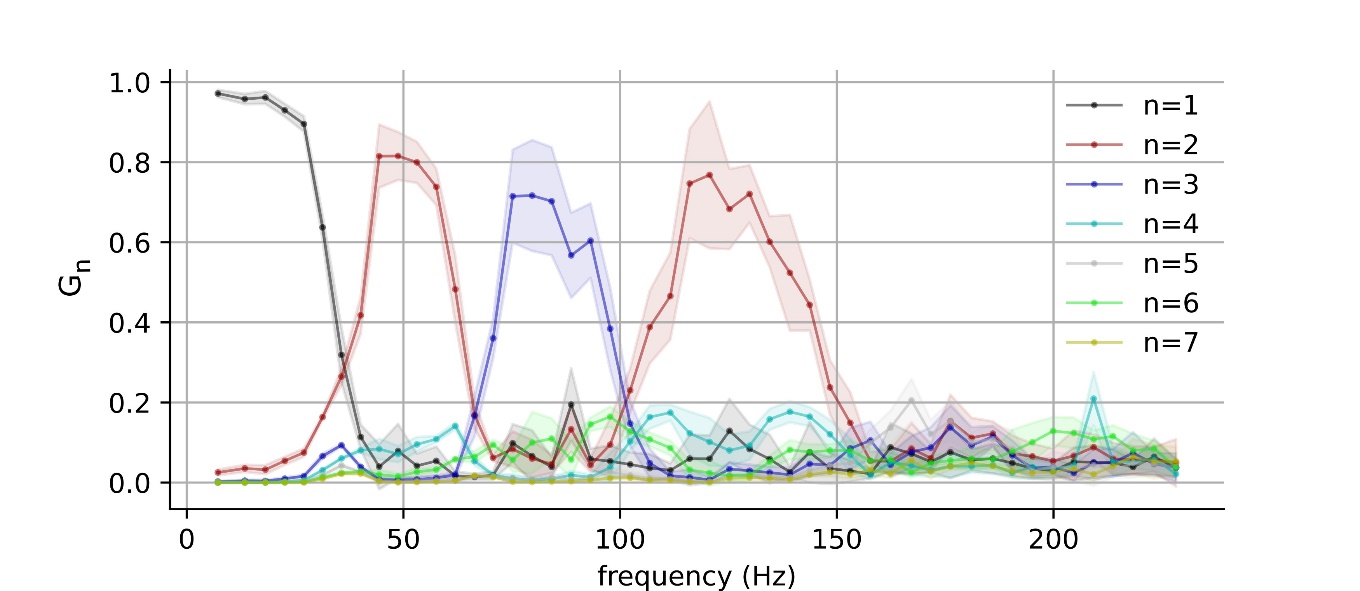


**Supplementary Figure 1.** The network-wide stability and the number of phase clusters in a larger population (N=500) of fast-spiking interneurons for increasing pyramidal neuron frequency. For each frequency, $G_{n}$ values were computed by randomly sampling 10 sets of 100 pairs of interneurons. Solid lines and shaded areas represent means and standard deviations respectively.


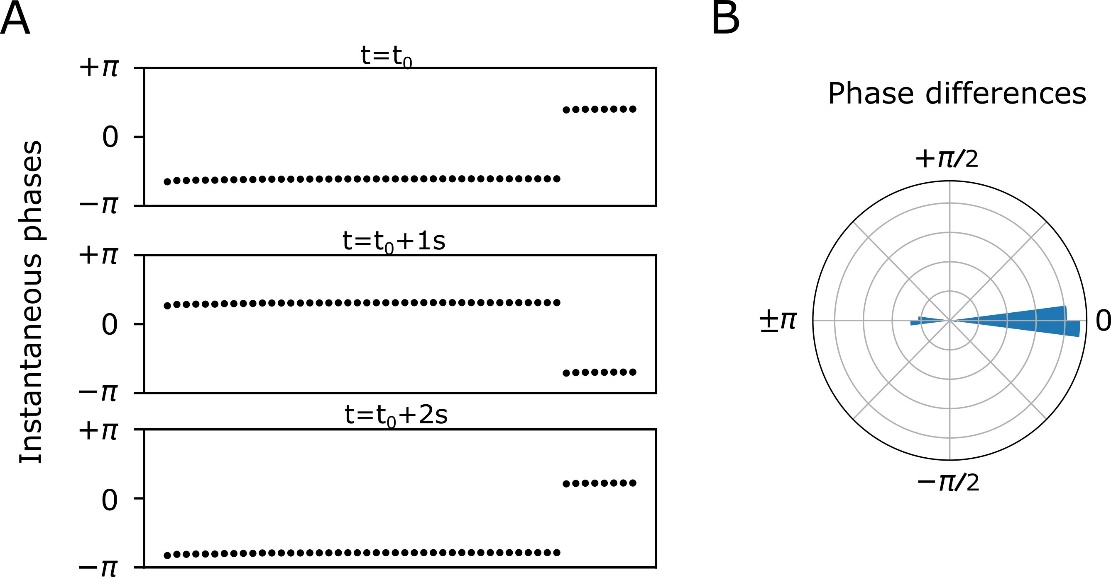


**Supplementary Figure 2. A.** Phase clusters are non-itinerant when the I-I connection probability is zero. Neurons (black dots) are ordered by their instantaneous phases in the top panel, and this ordering is preserved in the bottom two panels. Two phase clusters persist over time. However, the neurons remain in the same cluster over time, unlike the case when the I-I connection probability is non-zero (see **Figure 2**). **B.** Persistent phase relationships between neurons are illustrated in a polar histogram by plotting the phase differences between all distinct pairs of neurons.


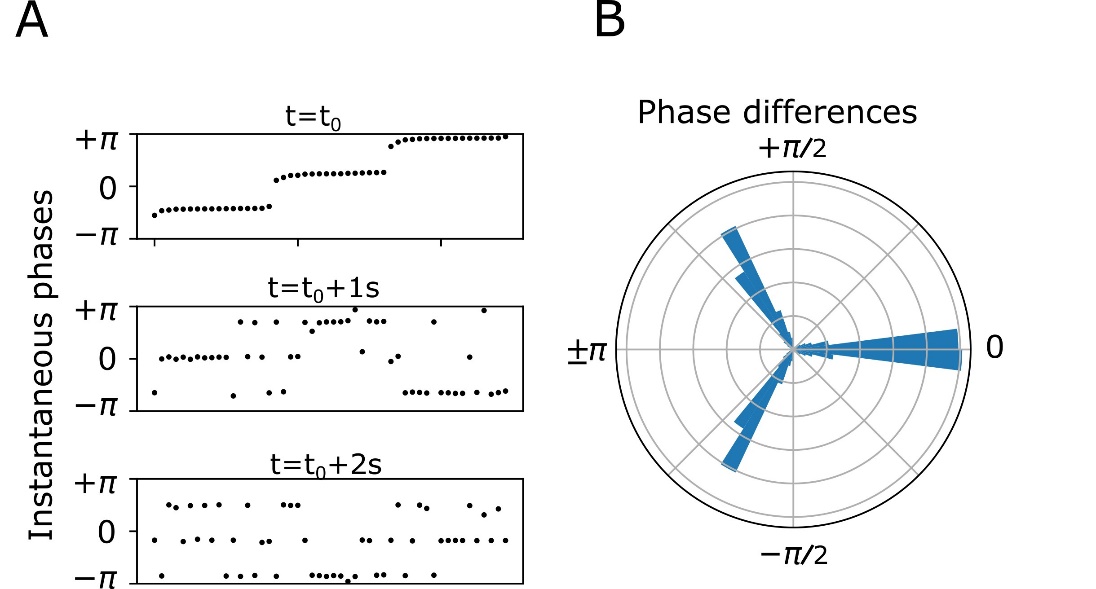


**Supplementary Figure 3. A.** Endogenous itinerancy characterized by phase clusters at three time points over a 2s duration. Neurons (black dots) are ordered by their instantaneous phases in the top panel, and this ordering is preserved in the bottom two panels. Three phase clusters persist over time, and individual neurons spontaneously switch from one cluster to the other. **B.** Persistent phase relationships between neurons are illustrated in a polar histogram by plotting the phase differences between all distinct pairs of neurons over the 2s duration.
